# Measuring social capital through multivariate analyses for the IQ-SC

**DOI:** 10.1186/s13104-015-0978-2

**Published:** 2015-01-20

**Authors:** Ana Cristina Viana Campos, Carolina Marques Borges, Andréa Maria Duarte Vargas, Viviane Elisangela Gomes, Simone Dutra Lucas, Efigênia Ferreira e Ferreira

**Affiliations:** School of Dentistry, Universidade Federal de Minas Gerais, Faculdade de Odontologia // Presidente Antônio Carlos 6627, Belo Horizonte, 31270-901 Minas Gerais Brazil; Department of Community and Preventive Dentistry, School of Dentistry, Universidade Federal de Minas Gerais, Presidente Antônio Carlos 6627, Belo Horizonte, 31270-901 Minas Gerais Brazil

**Keywords:** Social capital, Statistical analysis, Adolescent

## Abstract

**Background:**

Social capital can be viewed as a societal process that works toward the common good as well as toward the good of the collective based on trust, reciprocity, and solidarity.

Our study aimed to present two multivariate statistical analyses to examine the formation of latent classes of social capital using the IQ-SC and to identify the most important factors in building an indicator of individual social capital.

**Findings:**

A cross-sectional study was conducted in 2009 among working adolescents supported by a Brazilian NGO. The sample consisted of 363 individuals, and data were collected using the World Bank Questionnaire for measuring social capital. First, the participants were grouped by a segmentation analysis using the *Two Step Cluster* method based on the Euclidian distance and the *centroid* criteria as the criteria for aggregate answers. Using specific weights for each item, discriminant analysis was used to validate the cluster analysis in an attempt to maximize the variance among the groups with respect to the variance within the clusters. “Community participation” and “trust in one’s neighbors” contributed significantly to the development of the model with two distinct discriminant functions (p < 0.001). The majority of cases (95.0%) and non-cases (93.1%) were correctly classified by discriminant analysis.

**Conclusions:**

The two multivariate analyses (segmentation analysis and canonical discriminant analysis), used together, can be considered good choices for measuring social capital. Our results indicate that it is possible to form three social capital groups (low, medium and high) using the IQ-SC.

**Electronic supplementary material:**

The online version of this article (doi:10.1186/s13104-015-0978-2) contains supplementary material, which is available to authorized users.

## Background

The concept of social capital and its application is one of the most widespread lines of societal analysis as well as an alternative for integrating diverse areas of knowledge [1,2]. Social capital can be viewed as a societal process that works toward the common good and the good of the collective based on trust, reciprocity, and solidarity [3]. The aggregation of resources stemming from an association or group can also generate both individual and collective benefits, even over the long term [4]. Our study assumes that, in general, social capital refers to a system of networks, cohesion, trust, and relationships that promote collective actions based on common goals [3,5,6].

Social capital refers to the features of social structure that act as resources such as the levels of interpersonal trust and the norms of reciprocity, which can benefit individuals and facilitate collective actions [1,5]. Bain and Hicks [7] provided the first distinction between two different dimensions of social capital, introducing its structural and cognitive aspects. On the one hand, structural social capital (SSC) refers to the extension and intensity of associational links or, in a broad definition, refers to “what people do”. On the other hand, cognitive social capital (CSC) covers the sentiments, beliefs and values, referring to “what people think” [8]. The SSC can be subdivided into bonding, bridging and linking. Bonding social capital refers to connections among similar individuals sharing similar characteristics that stimulate mutual support (e.g., family). Bridging social capital refers to connections among people who are not alike (e.g., different religions and diverse socioeconomic backgrounds). Finally, linking social capital consists of trusting relationships between people who are interacting across power or authority [9].

Some studies have categorized the mechanisms linking social capital to health outcomes into macro and micro levels [10]. On the micro level, the possible pathways linking social capital and health are the influences on health-related behaviors such as physical activity [11] and on access to services and amenities that affect the psychosocial process [12], an example of which is the influence of workplace social capital on employee mental health [13]. With respect to the macro influence, social capital is related to the possibility of changes in the structure of communities based on health promotion policies [14], reducing income inequality [15] and increasing the number of public spaces to enhance the level of face-to-face contacts [16]. A recent literature review addressed the association between neighborhood social capital and the health/wellbeing status of children and adolescents. Social cohesion and neighborhood social control influence the impact of socio-economic factors on health outcomes in youth who reside in deprived areas [17].

Regarding the measurement of social capital, the World Bank developed a structured tool entitled the Integrated Questionnaire for the Measurement of Social Capital (IQ-SC), which is divided into 6 dimensions addressing 27 questions [18], as follows. *Groups and networks* is the dimension that considers the nature, extent and diversity of the participation of a member of a household in various types of social organizations and informal networks. The *trust and solidarity* dimension seeks to collect data on trust in neighbors, key service providers, and strangers, as well as to determine how these perceptions have changed over time. The dimension of *collective action* investigates how household members have worked on joint projects for the community. The *sources of information* dimension concerns the means by which poor households receive information about market conditions and public services, as well as where they have access to infrastructure communication. Considering that people in a community can be quite different, the questions regarding the *social cohesion and inclusion* dimension seek to identify the nature and size of these differences. In the last section, it is important to ascertain the *feeling of happiness* or the personal effectiveness and ability of household members to influence local events and broader policy responses [18].

The IQ-SC lacks a final component to measure the levels or degrees of individual social capital (e.g., low, medium, and high), which would facilitate the analysis and interpretation of results. Our study aimed to present two multivariate statistical analyses to examine the formation of latent classes of individuals by the IQ-SC, as well as to identify the most important factors in building an indicator of individual social capital. We hypothesize that discriminant and canonical discriminant analyses are adequate for assessing social capital measured by the World Bank Integrated Questionnaire for the measurement of social capital.

## Methods

This cross-sectional exploratory study included all working adolescents assisted by a Brazilian Non-Governmental Organization (NGO). The NGO was established in 1966 with the purpose of connecting working adolescents with local employers. The only criteria required by the NGO for working adolescents on applications were as follows: a) aged between 16 years and 17 years and 11 months, b) attending a public school, c) coming from low socioeconomic status, and d) completed the qualification course provided by the NGO. Neither race nor religious background was considered at admission. Sampling procedures, data collection and ethical issues were previously published elsewhere [19].

Data collection was conducted in January 2010 by administering a questionnaire to each participant. The majority of the 363 adolescents registered in the NGO during the data collection were male (95.9%). Therefore, we excluded the girls from the sample. The participants were those present during a special weekend meeting who had signed an informed consent form to participate in the survey and from whom we also obtained consent from their parents or guardians. Ethics approval was granted by the Research Ethics Committee at Universidade Federal de Minas Gerais (UFMG).

### Analysis

The IQ-MCS does not provide the users with a final score for measuring social capital [19]. Considering that this instrument is easy to apply and can be used in large epidemiological surveys, it is important to investigate a statistical method for analyzing the questionnaire results. Recommendations for analyses of the IQ-MCS have focused first on three dimensions (groups and networks, trust and solidarity, and collective action and cooperation), which are considered the basic indicators for measuring social capital to guide public policies [18]. The present study used these three dimensions concurrently to identify the most important factors in building an indicator of individual social capital for the studied population.

### Segmentation analysis

We opted for segmentation analysis to measure the social capital of participants because it allows for an initial selection of variables and random allocation. Considering that the sample is relatively small and homogeneous, we can simultaneously optimize the analysis to determine the best allocation of participants within groups. This analysis is an analytical statistics tool used to define the development of mutually exclusive significant subgroups based on the similarities among individuals, but it did not use prior knowledge of the allocation within the groups. When the grouping of the data is successful, the groups are internally homogeneous with high external heterogeneity [20].

The *Two-Step Cluster* was used to group the sample according to the similarities in the participants’ answers. This method has two phases; the participants were grouped into small subgroups by the shortest distance and then into a desired number of clusters, which was automatically selected by the program. An advantage of this method is the possibility of simultaneously manipulating the continuous and categorical variables formed; additionally, the cluster number is automatically determined [21]. The definition of the applied algorithm allows for the following requirements for selecting the desired number of clusters and characteristics: 1) select an item from each dimension of the social capital instrument; 2) opt for items that maintain the theoretical coherence of the instrument; 3) obtain a smaller possible number of excluded cases; and 4) create four distinct clusters in at least one characteristic.

Three distinct clusters were automatically created. For the present study, the number of close friends (item 06) was selected as the *centroid* of the clusters because it was a continuous item. The other two items were related to trust in one’s neighbors (item 09) and community participation (item 12).

At the end of *Two-Step Cluster*, we obtained a variable called “social capital” that was classified, as in other studies [6,17,22], in ascending order into the following three categories: low, medium and high. As a result, the cluster with high social capital would be one that would bring together adolescents with more positive features in each item, i.e., greater number of close friends, greatest trust in a neighbor, and greater participation in community activities.

### Discriminant analysis

The discriminant analysis was used to validate the described segmentation analysis. The discrimination is performed by estimating the weights of each variable, with the goal of maximizing the variance between and within groups so that the groups stand out as much as possible for the values of the discriminant function. In the analysis, we multiply each independent variable by its corresponding weight and then total these products; the result is a discriminant value for each subject considered in the analysis [23].

The three initial dimensions of the IQ-SC included a total of 14 items. However, the “name of group”, “social characteristics of the group members”, “education and occupation of members of the group” and “social interaction group” either could or could not be answered. These four items were excluded from the discriminant analysis because they did not contribute to separating the adolescents into clusters or because their exclusion minimized the level of missing data. Eleven independent items were selected, as follows: number of groups, number of close friends, financial aid, overall trust, trust in one’s neighbors, trust in the local government, trust in the central government, time dedicated to community projects, money to contribute to community projects, community participation, and cooperation among the community members.

The first step of the discriminant analysis is to randomly split the sample into two subgroups: the original analysis sample and the test sample. This procedure, known as cross-validation, is the most commonly used in the literature. The cross-validation has a discriminant function in a subgroup of the participants in the sample (original analysis sample), as well as in the testing of a second subgroup (test sample) [20]. The total sample of people who responded to the questionnaire (N = 363) was divided into two groups using a statistics program, creating one sample for analysis consisting of 70% (n_1_ = 254) of the adolescents and another test sample with the remaining 30% (n_2_ = 109).

The second step is to choose the variables that assess the formed discriminant function. For this, a bivariate F test and the *Wilks’ Lambda* test were used to measure the potential contribution of each independent item for separating the cases. Next, the discriminant linear function is constructed using coefficients that allow one to understand the value of the score from the analyzed data. Information about the relative effectiveness of each discriminant function is provided by a table of *eigenvalues*. Each function can be represented by the following equation: *Z = a + W*_*1*_*x*_*1*_ *+ … + w*_*2*_*X*_*2*_*w*_*n*_*X*_*n*_, in which *Z* represents the score of the discriminant function, *a* is the discriminant coefficient, and *w* is the weight discriminant for the independent variable *X* and the value of each independent variable.

Finally, internal validation of the results is obtained in the discriminant analysis to verify the efficiency of classification of the original observations and cross-validation. This method produces linear combinations of independent variables that best discriminate groups established by the dependent variable, defining the rules for classifying the elements in each group. The classification of the original cases, validated and unselected, is performed by separately calculating the percentage of the total correctly classified cases by the total cases.

This study chose to use a method that simultaneously evaluates all items. This means that the program analyzes cases (responses of adolescents to the IQ-SC) one by one and then allocates them in such a way that the best possible graphical separation between the groups is created. The construction of the databank was performed using the *Statistical Package for Social Sciences for Windows* (SPSS), version 17, for data analysis, at a significance level of 0.05 for all tests within the study.

## Results

Descriptive analysis showed that of the total respondents (N = 363), 50.7% were 16 years old, 95.9% were male, 88.7% of the adolescents were enrolled in the second grade of Brazilian high school, and 13.8% declared their skin color as white. According to segmentation analysis, 111 (31.3%) adolescents were gathered into a high social capital group (cluster 1), 143 (40.3%) into an intermediate social capital group (cluster 2), and 101 (28.5%) into a low social capital group (cluster 3). Only eight adolescents (2.2%) were not classified into any cluster (Table 1).

**Table 1:** **Distribution of adolescents in clusters through segmentation analysis for the IQ-SC, Brazil, 2010**

**Item answers**	**Clusters**						**Total**	
	**High (N = 111)**		**Medium (N = 143)**		**Low (N = 101)**			
	**n**	**%**	**n**	**%**	**n**	**%**	**n**	**%**
Trust in one’s neighbors								
Yes	54	48.6	92	64.3	12	11.9	158	44.5
Maybe	29	26.1	51	35.7	00	0,0	80	22.5
No	28	25.2	00	0.0	89	88.1	117	32.3
Work for the benefit of the community								
Yes	107	96.4	00	0.0	00	0.0	107	30.1
No	04	3.6	143	100.0	101	100.0	248	69.8
Friends*	13.9 (±29.2)		7.0 (±6.8)		6.3 (±7.8)		8.9 (±17.5)	

*Values presented as the mean and standard deviation.

In the discriminant analysis, the program created two linear discriminant functions (here called Z_1_ and Z_2_) to validate the segmentation analysis. The results from the F bivariate test demonstrated that, at a significance level of 5%, the items of “community participation” and “trust in one’s neighbors” contributed significantly toward the creation of the model (p < 0.001). The item “community participation” contributed 93.9% toward the creation of discriminant linear function 1, whereas “trust in one’s neighbors” contributed 82.8% toward the creation of discriminant linear function 2 [see Additional file 1].

The test for measuring the discriminant global significance demonstrated that the analysis carried out with the eleven items was statistically significant for both formulated functions (p < 0.001). The *eigenvalue* was 54.15 for function 1 and 1.02 for function 2, given that the canonical correlation in the first function explains 99.1% (R^2^ = 0.991) of the discrimination among the clusters [see Additional file 2].

Table 2 contains information on the classification of the observations within the respective clusters, considering that the great majority of the cases (95.0%) and non-cases (93.1%) were correctly classified by the discriminant analysis. Of the 72 cases that were pre-classified within cluster 1 (high social capital), 71 kept that classification, which could be considered a positive discriminating result for this cluster compared with the others. Within clusters 2 and 3, there was an exchange allocation in 10 cases, representing 5.7% and 8.3% classification errors, respectively.

**Table 2:** **Classification of the clusters according to the discriminant analysis for the IQ-SC, Brazil, 2010**

**Cluster**				**Predicted group membership**			**Total**
				**High**	**Medium**	**Low**	
Selected cases	Original^a^	N	High	71	01	-	72
			Medium	-	83	05	88
			Low	-	05	55	60
		%	High	98.6	1.4	-	100.0
			Medium	-	94.3	5.7	100.0
			Low	-	8.3	91.7	100.0
	Cross-validated^b^	N	High	71	01	-	72
			Medium	-	82	06	88
			Low	-	05	55	60
		%	High	98.6	1.4	-	100.0
			Medium	-	93.2	6.8	100.0
			Low	-	8.3	91.7	100.0
Unselected cases	Original^c^	N	High	28	01	02	31
			Medium	-	38	02	40
			Low	-	02	28	30
		%	High	90.3	3.2	6.5	100.0
			Medium	-	95.0	5.0	100.0
			Low	-	6.7	93.3	100.0

^a^95.0% of the original selected cases were correctly classified.

^b^93.1% of the cross-validated selected cases were correctly classified.

^c^94.5% of the unselected original cases were correctly classified.

The final model for the present study consisted of two discriminant functions by means of linear combinations of independent items to provide the most appropriate discrimination among the clusters of social capital. According to these results, the sum of the two discriminant functions created by the analysis (Z_1_ and Z_2_) provides a statistical measure of the IQ-SC social capital for each adolescent. From this score, each adolescent was allocated into the low, medium or high capital group [see Additional file 3]. A scatter plot graphically represents the relationship between the discriminant functions and clusters [see Additional file 4]. The first function, shown on the horizontal axis, separates cluster 1 (high social capital) from the others. The second function separates clusters 2 and 3; however, the proximity of the *centroids* of these two clusters suggests that the separation between the two is rather weak.

## Discussion

The potential effects of social capital on adolescents’ behaviors or health outcomes have been documented in the scientific literature. Gillespie [24] examined the relationship between geographic mobility, adolescent academic achievement and behavior problems, considering that social capital factors moderate the effects of moving on behavior but not achievement. A recent study investigated the association between individual and school-level social capital and the student body mass index (BMI) and reported that boys attending a school with high “treatment” had an inverse association with BMI. They suggested enhancing school social capital as a novel approach for addressing student obesity [25].

Unlike other studies that used the World Bank Integrated Questionnaire for measuring social capital [6,19,21], the focus of this study was to propose a statistical method (segmentation analysis followed by canonical discriminant analysis) to form social capital groups of adolescents. Therefore, the discussion of this study will be directed by the method rather than by a conceptual evaluation of the results. The World Bank organized the IQ-SC with the aim of providing a set of key information for all of those interested in generating quantitative data on various dimensions of social capital. The IQ-SC is based on measuring the social capital at both the household and individual levels. The participation and density of associations in the household, levels of trust, and collective action are good indicators of social capital stocks. Therefore, these should be concurrently calculated and analyzed [18,26]. Although we have not examined the psychometric properties of the underlying IQ-SC, our study represents one of the first efforts to provide a method of analysis for the IQ-MS.

Two-step cluster analysis is based on the Euclidean distance classifier. The distance between two participants (*i* and *j*) is the square root of the sum of squares of differences between the values *i* and *j* for all variables [20]. Adolescents who are closest to the centroid that gathers the variables under analysis will be placed in the same group. In our study, only eight participants were not allocated to any of the three groups of social capital. A lower absence of data indicates a better match of the segmentation analysis, i.e., allocation into the three groups is a good measure of capital for this sample. Therefore, segmentation analysis seems to be appropriate and insightful for determining the best way to analyze the IQ-SC through the formation of groups with social capital levels.

Our results demonstrated the construction of two discriminant functions that allocates every teenager in the bottom group, medium or high capital. The discriminant analysis is mainly used to understand the differences among the clusters and to predict the probability that a participant will pertain to one cluster in particular, which is based on the most appropriate items for this separation. Additionally, the intention is to generate a model that offers a clear direction about predictable classification cases and their respective weights [20].

The parameters for classifying the discriminant functions were calculated by testing the probability of the correct allocation of adolescents in three clusters of social capital. The apparent rate of correct classification was high (95.0%), which illustrates the efficiency of the model used for allocating the adolescents into clusters with low, medium, and high social capital. This appears to be a good division for the linear distribution of the clusters by means of discriminant functions, given that the first function could separate cluster 1 (high social capital) from the other clusters with high precision (99.1%).

The analysis of the most important items for each function provided theoretical plausibility and applicability of the items as indicators of social capital stocks. The tests carried out in this study highlighted statistically significant differences among the clusters. The discriminant functions attributed weights to the most important items in this separation. The segmentation by means of the three items (“number of close friends”, “community participation”, and “trust in one’s neighbors”) provided a good indicator for measuring capital in the investigated population by the creation of three distinct clusters. With respect to discriminant function 1, “community participation” contributed greatly toward separating cluster 1 from the others because its coefficients are larger in absolute terms (93.9%). These results indicate that the adolescents with high social capital are, in general, the most active in the community to which they belong.

Social capital is based on the non-monetary precursors for the creation of bonds, trust, and social support [3]. This may be a possible explanation for the positive effect of community participation on measurement of the social capital of participants. In the present study, the item related to trust in one’s neighbors played a key role in the construction of the second discriminant function (82.8%) and presented with the second lowest Lambda value (0.574) and the highest F value (80.554). During adolescence, there is a certain natural distancing between parents and their children; friends become the adolescents’ confidants and companions. Extending this rationale to the neighborhood, one can imagine that trust in one’s neighbors is intimately linked to the formation of friendship bonds among adolescents [27-29].

For the present study, friendship was decisive in the separation of clusters during the segmentation analysis, especially in the creation of the high social capital group. As it is a continuous item, it can be presumed that this item has a certain power of separation due to the dissimilarities among adolescents, given that the analysis of segmentation provides the average and standard deviation for this type of data. However, in the discriminant analysis, this finding was not confirmed because the coefficients were low for both functions 1 (−0.021) and 2 (0.010), suggesting that the average social capital cannot be explained by one sole item. Studies indicate that adolescents consider themselves good friends; they have no problem making friends and have a positive self-image about their relationships with friends [30,31].

Lastly, the results confirmed our hypothesis that segmentation analysis and discriminant analysis are good methods for computing the data from the IQ-SC. The function discriminants create an equation that will minimize the possibility of misclassifying cases into their respective groups [12]. A scatter plot graphically shows the relatively large distances between the centroids of each social capital group, with a greater efficiency in discriminating the high social capital group [see Additional file 4]. Using eleven items of the IQ-SC, it was possible to combine (weigh) the variable scores so that a single new composite variable, the discriminant score, was produced. Therefore, the analysis uses the responses of the selected items to compute a statistical measure of social capital for each participant to directly rank the participants into one of the groups of low, medium or high capital.

### Study limitations

The first limitation of the present study is the measurement of the social capital in a homogeneous sample with respect to the socioeconomic conditions and perceptions. One possible explanation for this constraint is that the particular characteristics of this group of adolescents (the number of participants in this study, as well as the low variability in age, sex, and socioeconomic conditions) may have influenced the creation of the cluster. Another point is that 95% of adolescents assisted by the NGO were men, which is why we excluded the only three girls enrolled in NGO activities. Therefore, future studies using the IQ-SC should most likely be carried out at the population level; the population should have as high a level of heterogeneity as possible to better clarify the use of these analyses in measuring social capital and in choosing the most appropriate statistical treatment of the items of this instrument.

The IQ-SC was proposed in six dimensions that represent the main factors related to the concept of social capital. During data analysis, we understood that the social capital could be considered a latent variable, i.e., something that is not directly measurable. When we performed the confirmatory factor analysis, the exploratory analysis indicated the inadequacy of questions for factorization. This is likely because the questionnaire has already been organized into six dimensions. Therefore, it would make sense to create new dimensions.

With respect to discriminant analysis, it is important to highlight that the results should be analyzed cautiously, considering that the same items used for the segmentation analysis were also the most discriminant. However, all possible combinations within the segmentation analysis were previously contemplated in an attempt to choose the items that best separate into the clusters according to the criteria described above. Considering that this is an exploratory study, the most important action was to define the weight of each item in the separation of the clusters.

Measuring social capital using the IQ-SC is a challenge that warrants further study, preferably at the population level, to better propose and clarify the use of multivariate analyses in the measurement of social capital when using this instrument.

## Conclusion

In the present study, the two multivariate analyses (segmentation analysis and canonical discriminant analysis), when used together, can be considered good choices for measuring social capital. Our results indicate that it is possible to generate a good, unique statistical measure for the IQ-SC by forming three groups of social capital (low, medium and high).

The results of the discriminant analysis indicate that the number of friends, community participation and trust in neighbors for this sample had a higher influence on the other variables, confirming that the dimensions relating to groups and networks, trust and solidarity and collective action are determinant factors of social capital.

The other items were considered less important in the analysis because they did not significantly contribute to the separation of groups. Moreover, these results suggest that social capital cannot be explained by a single item or by causal item associations. By measuring the social capital of adolescents for the 11 items of the IQ-SC, only two functions remained after constructing the canonical model. This means that the similar ratio associated with the total power of discrimination between the groups was maintained in the reduction of the Euclidean space compared to the dimensions.

This study helps to emphasize the importance of statistically and non-randomly choosing a cohesive set of questionnaire items that can measure social capital in all dimensions proposed by the World Bank.

We hope that future studies using the IQ-SC will confirm the importance of the selected items as well as identify other favorable indicators for measuring social capital, especially in homogeneous samples. Furthermore, this model can be applied to measure the capital of other populations as well as serve as the starting point for further analysis.

## Additional files

Additional file 1:
**Equality and structured matrix tests for each variable and discriminant function.**
Additional file 2:
**Summary of the canonical discriminant function for the variables, with results from the Wilks’ Lambda test for each discriminant function.**
Additional file 3:
**System of equations for each discriminant function.**
Additional file 4:
**Cluster 1: High Social Capital.** Cluster 2: Medium Social Capital. Cluster 3: Low Social Capital. Group Centroid.

## Abbreviations

NGO: Non-governmental organization
SC-QI: Integrated questionnaire for the measurement of social capital
