# Supplementary material for: Measuring social capital through multivariate analyses for the IQ-SC
Source: BMC Res Notes. 2015 Jan 20;8:11. doi: 10.1186/s13104-015-0978-2 (PMC4304630; doi:10.1186/s13104-015-0978-2)
Supplement: Additional file 1: — Equality and structured matrix tests for each variable and discriminant function. [file 13104_2015_978_MOESM1_ESM.pdf]

Additional file 1. Equality and structured matrix tests for each variable and discriminant function.

Variables	Discriminant function		Wilks' Lambda	F	P
	1	2			
X_{10}: community participation	0.939*	-0.066	0.021	5182.355	<0.001
X_1: number of groups	-0.021*	-0.018	0.977	2.603	0.076
X_2: number of friends	-0.019*	-0.001	0.981	2.104	0.124
X_5: trust in one's neighbors	0.027	0.828*	0.574	80.554	<0.001
X_4: overall trust	-0.004	-0.142*	0.979	2.338	0.099
X_{11}: cooperation	0.011	0.142*	0.973	2.954	0.054
X_9: money	0.010	0.098*	0.985	1.622	0.200
X_7: central government	0.015	0.088*	0.981	2.126	0.122
X_3: financial aid	0.008	-0.072*	0.991	0.970	0.381
X_6: local government	0.009	0.044*	0.994	0.682	0.506
X_8: time	0.002	-0.014*	1.000	0.038	0.963

* Greatest absolute correlation between the variable and the respective discriminant function
