# Supplementary material for: Measuring social capital through multivariate analyses for the IQ-SC
Source: BMC Res Notes. 2015 Jan 20;8:11. doi: 10.1186/s13104-015-0978-2 (PMC4304630; doi:10.1186/s13104-015-0978-2)
Supplement: Additional file 2: — Summary of the canonical discriminant function for the variables, with results from the Wilks’ Lambda test for each discriminant function. [file 13104_2015_978_MOESM2_ESM.pdf]

Additional file 2. Summary of the canonical discriminant function for the variables with results from the Wilks' Lambda test for each discriminant function.

**Variables**	**Discriminant function***	
	**1**	**2**
X_1: number of groups	0.000	0.008
X_2: number of friends	-0.021	0.010
X_3: financial aid	-0.070	-0.202
X_4: trust	-0.175	-2.389
X_5: trust in one's neighbors	0.088	1.264
X_6: local government	0.033	-0.215
X_7: central government	-0.007	0.073
X_8: time	-0.055	-0.097
X_9: money	0.128	0.218
X_{10}: community participation	15.673	-0.437
X_{11}: cooperation	-0.059	0.053
(Constant)	-25.880	2.281

**Canonical discriminant function**		
Self-worth	54.150	1.024
% variance	98.1	1.9
Canonical Correlation (R^2)	0.991	0.711
Wilks' Lambda Test	0.009	0.494
p-value	<0.001	<0.001

* Non-standardized coefficients of the canonical discriminant function.
