# Supplementary material for: Measuring social capital through multivariate analyses for the IQ-SC
Source: BMC Res Notes. 2015 Jan 20;8:11. doi: 10.1186/s13104-015-0978-2 (PMC4304630; doi:10.1186/s13104-015-0978-2)
Supplement: Additional file 3: — System of equations for each discriminant function. [file 13104_2015_978_MOESM3_ESM.pdf]

Additional file 3. System of equations for the discriminant functions.

$$\begin{cases} Z_1 = -25.880 + (-0.021)X_2 + (-0.070)X_3 + (-0.175)X_4 + (0.088)X_5 + (0.033)X_6 + \\ (-0.007)X_7 + (-0.055)X_8 + (0.128)X_9 + (15.673)X_{10} + (-0.059)X_{11} \\ \\ Z_2 = 2.281 + (-0.008)X_1 \ (0.010)X_2 + (-0.202)X_3 + (-2.389)X_4 + (1.264)X_5 + \\ (-0.215)X_6 + (0.073)X_7 + (-0.097)X_8 + (0.218)X_9 + (-0.0437)X_{10} + (0.053)X_{11} \end{cases}$$

Legend:

Z_1: Function 1

Z_2: Function 2

X_1: number of groups

X_2: number of friends

X_3: financial aid

X_4: trust

X_5: trust in one’s neighbors

X_6: local government

X_7: central government

X_8: time

X_9: money

X_{10}: community participation

X_{11}: cooperation
