# Supplementary figures and images for: Measuring social capital through multivariate analyses for the IQ-SC
Source: BMC Res Notes. 2015 Jan 20;8:11. doi: 10.1186/s13104-015-0978-2 (PMC4304630; doi:10.1186/s13104-015-0978-2)

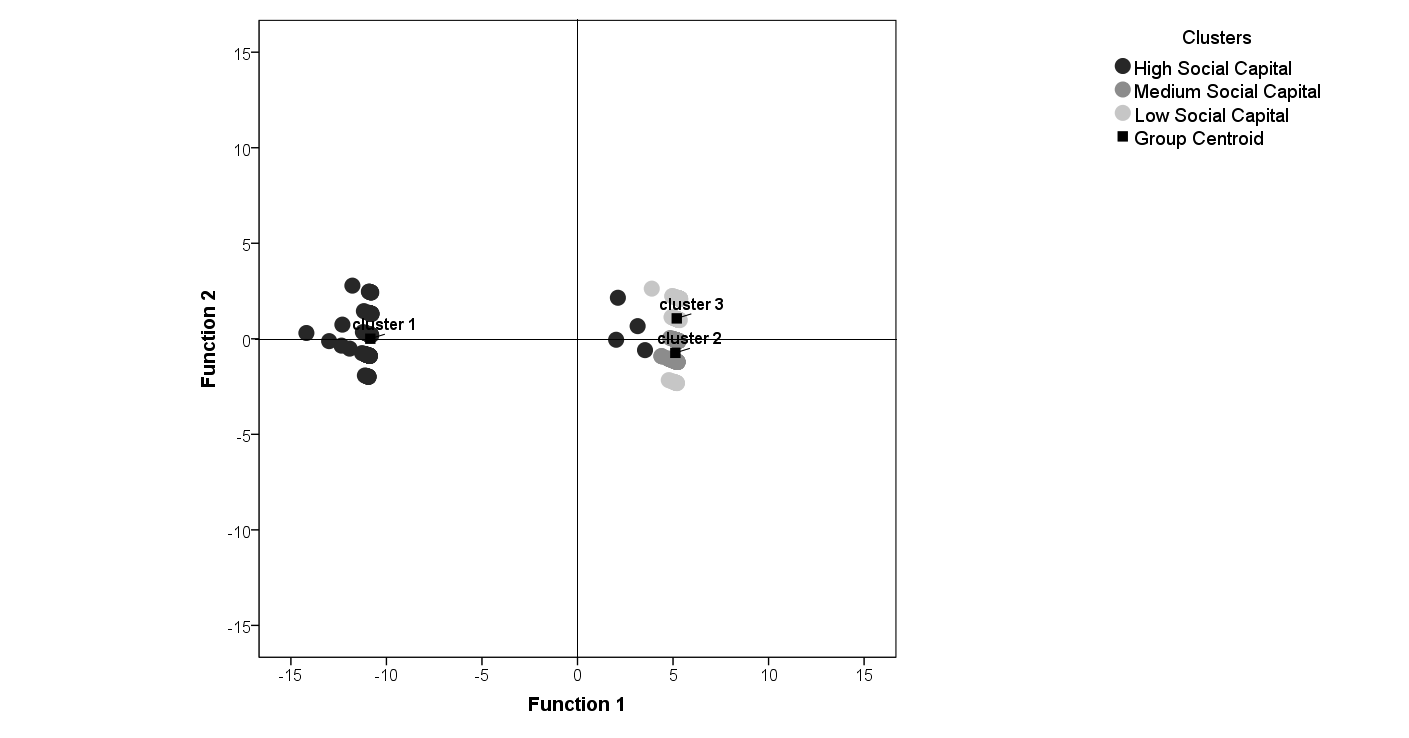

Supplement: Additional file 4: — Cluster 1: High Social Capital. Cluster 2: Medium Social Capital. Cluster 3: Low Social Capital. Group Centroid. [file 13104_2015_978_MOESM4_ESM.png]
